# Pharmacotherapy for social anxiety disorder: An evidence-based treatment review

**DOI:** 10.1017/S1092852926101254

**Published:** 2026-10-16

**Authors:** Jeffrey Robert Strawn, Stephen M. Stahl, Azmi Nasser, Andrew J. Cutler, Márcio Bernik

**Affiliations:** 1 Department of Psychiatry & Behavioral Neuroscience, University of Cincinnati College of Medicine, Cincinnati, OH, USA; 2 Neuroscience Education Institute, USA; 3 Department of Psychiatry, University of California San Diego, USA; 4 VistaGen Therapeutics Inc, San Francisco, CA, USA; 5 Psychiatry, SUNY Upstate Medical University, USA; 6 Anxiety Disorders Program, Institute of Psychiatry, University of Sao Paulo Medical School, Brazil

**Keywords:** Selective serotonin reuptake inhibitor, selective norepinephrine reuptake inhibitor, antidepressant, benzodiazepine, MAOIs

## Abstract

Social anxiety disorder (SAD) is a common and often chronic psychiatric illness characterized by persistent fear of social or performance situations and associated functional impairment and avoidance. Although multiple pharmacologic treatments have been studied, questions remain regarding optimal sequencing of therapies, long-term treatment strategies, and the management of partial or treatment-resistant illness. This narrative review synthesizes the evidence for pharmacologic treatments for SAD across medication classes, including antidepressants, benzodiazepines, monoamine oxidase inhibitors, GABA-related agents, second-generation antipsychotics, and emerging interventions. It also examines as-needed (PRN) interventions and steroid-based approaches, deprescribing and proposes a definition of treatment-resistant SAD. Randomized controlled trials, open-label studies, and guideline recommendations were reviewed to provide a framework for pharmacologic decision-making. Selective serotonin reuptake inhibitors (SSRIs) and serotonin–norepinephrine reuptake inhibitors (SNRIs) remain the best-supported first-line pharmacotherapies for SAD. Evidence supports switching within or across antidepressant classes and integrating pharmacotherapy with behavioral interventions in patients with incomplete response. Additional treatments, including benzodiazepines, monoamine oxidase inhibitors, pregabalin, and selected augmentation strategies, may be considered for patients with persistent symptoms, although the evidence base is more limited. Data regarding long-term outcomes and treatment-resistant populations remain relatively sparse. While serotonergic antidepressants remain the cornerstone of treatment, optimal management requires individualized sequencing of therapies, integration with behavioral interventions, and consideration of alternative mechanisms in patients with incomplete response. Future studies should prioritize comparative effectiveness, long-term outcomes, and interventions for treatment-resistant SAD.

## Introduction

Social anxiety disorder (SAD), formerly social phobia, is a common, chronic, and frequently disabling condition that typically emerges during adolescence or early adulthood.^1^ It is highly heritable^2^ and frequently persists throughout the lifespan.^1^ SAD is characterized by persistent fear of social or performance situations involving potential scrutiny by others and affects approximately 3–7% of adults and up to 5% of adolescents in community samples.^3^^,^^4^ The disorder is associated with substantial functional impairment across academic, occupational, and interpersonal domains.^5^ Beyond this functional impairment, SAD increases the risk of developing secondary psychiatric disorders, including major depressive disorder,^6^^–^^8^ substance use disorders, alcohol use disorder, and other anxiety disorders.^9^^,^^10^ SAD typically precedes the development of other disorders.^10^^,^^11^ SAD is also associated with suicidal ideation and behavior, which may be driven by interpersonal factors.^12^ Social disconnection and fear of negative evaluation may contribute to suicidality by increasing perceived burdensomeness and decreasing individuals’ sense of belongingness.^13^ Epidemiologic data further suggest that the increased risk of suicidality in SAD is not fully explained by comorbid mood disorders^14^ and may be related to the severity of social anxiety symptoms.^15^

Over the past four decades, substantial progress has been made in the systematic evaluation of pharmacologic treatments for SAD. Randomized controlled trials, open-label studies, and other clinical data support the efficacy of several medication classes, most commonly selective serotonin reuptake inhibitors (SSRIs) and serotonin–norepinephrine reuptake inhibitors (SNRIs). Additional agents, of which many are used “off label” (e.g., benzodiazepines, monoamine oxidase inhibitors, tricyclic antidepressants, beta-adrenergic antagonists, and other adjunctive interventions) have also been evaluated, although the strength and consistency of evidence vary substantially across classes and across individual agents, and relatively few of these have FDA approval for the treatment of SAD. These studies have helped establish serotonergic antidepressants as foundational pharmacologic treatments for SAD, yet important questions remain regarding comparative effectiveness, optimal sequencing and adjunctive use of therapies, and the management of partial or treatment-resistant illness.

Current international guidelines largely converge in recommending SSRIs and SNRIs as first-line pharmacologic treatments for SAD, although they differ somewhat in emphasis.^16^^-^^18^ The WFSBP guideline identifies escitalopram, fluvoxamine, paroxetine, sertraline, and venlafaxine as leading evidence-based options.^19^ Canadian and Japanese guidance similarly suggest SSRIs and venlafaxine as first-line pharmacotherapies, although the Japanese Society of Anxiety and Related Disorders guideline (published more than a decade ago) notes that the overall strength of evidence supporting these recommendations remains modest.^16^ In contrast, the Korean clinical consensus guideline highlights a somewhat broader set of first-line strategies in real-world practice, including SSRI or SNRI monotherapy as well as combination approaches incorporating beta-blockers or benzodiazepines for selected patients. Finally, the most recent guidelines to be published (Brazilian Psychiatric Association) recommend SSRIs as first-line treatment for SAD “due to their large effect size and database of evidence.”^20^ These guidelines recommend monoamine oxidase inhibitors, benzodiazepines, and the anticonvulsants pregabalin and gabapentin as additional agents with substantial evidence, but note “divergent results have been found for the serotonin-norepinephrine reuptake inhibitor venlafaxine,” which lowers their recommendation for such agents.^20^ Together, these guidelines underscore general agreement regarding the central role of serotonergic antidepressants while highlighting uncertainty regarding treatment sequencing, combination strategies, and the management of more complex presentations.

Despite the accumulation of randomized trials and the consensus among treatment guidelines, several important gaps remain in the pharmacotherapy literature for SAD. Many studies have focused primarily on short-term efficacy against placebo rather than comparative effectiveness across active agents or long-term outcomes such as relapse prevention and durability of response. In addition, relatively few trials have examined pharmacologic strategies for patients with partial response, common comorbidities, or treatment-resistant illness. These limitations are particularly relevant clinically, as patients frequently present with longstanding symptoms, comorbid disorders, or prior treatment exposure or having been unable to tolerate first-line medications. Accordingly, in the Harvard Brown Anxiety Research Project, only 35% of patients followed at these institutions recovered after 10 years of prospective follow-up.^21^^,^^22^

The aim of this narrative review is twofold. First, we synthesize the current evidence regarding pharmacotherapy for SAD. Second, we provide an expert synthesis of the evidence and identify key unmet needs in the treatment of SAD, including the management of partial or treatment-resistant illness, the role of combination strategies, and priorities for future clinical investigation. By integrating evidence from clinical trials, guideline recommendations, and emerging research directions, we aim to provide clinicians with a clearer framework for pharmacologic decision-making while highlighting areas where further study is urgently needed.

Accordingly, the pharmacologic literature for SAD is considered beginning with the serotonergic antidepressants, which have been the most extensively studied and remain the cornerstone of pharmacologic treatment across contemporary guidelines.^16^^-^^18^ Among these, selective serotonin reuptake inhibitors (SSRIs) were the first class of medications to demonstrate consistent efficacy in randomized trials and have since been evaluated across multiple formulations, dosing strategies, and clinical populations. The sections that follow review the evidence for individual antidepressants used in the treatment of SAD, beginning with SSRIs and subsequently addressing SNRIs and other pharmacologic approaches.

## Selective serotonin reuptake inhibitors

### Citalopram and escitalopram

Citalopram and its pharmacologically active S-enantiomer, escitalopram, are highly selective serotonin transporter inhibitors with minimal activity for other receptor systems. Citalopram was introduced in the United States in 1998, followed by escitalopram in 2002. Escitalopram represents the active stereoisomer responsible for most of citalopram’s serotonergic activity and is among the most selective inhibitors of the serotonin transporter within the SSRI class.

Evidence supporting citalopram in SAD is limited but suggests potential efficacy. In an 8-week randomized, open-label, rater-blinded trial comparing citalopram with the reversible monoamine oxidase inhibitor moclobemide in patients with social phobia, response rates were similar between the two treatments (approximately 75% for citalopram and 74% for moclobemide), defined as a ≥ 50% reduction in LSAS scores and a rating of “much” or “very much improved” on the CGI-I scale.^23^ Neuroimaging studies also demonstrated that successful citalopram treatment decreased regional cerebral blood flow responses within limbic regions, including the amygdala and hippocampus, during social threat paradigms.^24^

Escitalopram has been more extensively studied in SAD. In a large randomized, double-blind, placebo-controlled trial, escitalopram (10–20 mg/day) produced significantly greater reductions in LSAS scores compared with placebo and higher response rates (54% versus 39%) over 12 weeks of treatment.^25^ Longer-term studies have similarly demonstrated sustained efficacy and good tolerability. In a 24-week randomized trial that included paroxetine as an active reference arm (not powered for formal between-drug comparisons), escitalopram (5–20 mg/day) was associated with significantly greater improvement in LSAS scores compared with placebo, with continued improvement over the treatment period and response rates exceeding placebo across doses.^26^ Additional randomized controlled data in Japanese patients with SAD have confirmed the efficacy and tolerability of escitalopram at doses of 10–20 mg/day, further supporting its effectiveness across patient populations.^27^

Taken together, these findings support escitalopram as an effective and well-tolerated SSRI for the treatment of SAD, with multiple randomized controlled trials demonstrating both short- and longer-term benefit. In contrast, evidence for citalopram is more limited and derives primarily from smaller comparative and mechanistic studies.

### Fluvoxamine

Fluvoxamine is a potent serotonin transporter inhibitor that also acts as a sigma-1 receptor agonist, a mechanism implicated in modulation of multiple transmitters, inflammation, stress circuitry, and synaptic plasticity. Pharmacokinetically, fluvoxamine has high CNS penetration, with spectroscopy studies suggesting that brain concentrations substantially exceed plasma levels (approximately 20-fold higher at steady state), with relatively rapid CNS steady state.^28^ It has been studied extensively in SAD and is FDA-approved for this condition in adults. In one of the first studies, a 12-week double-blind study of 30 patients, fluvoxamine (150 mg/day) produced substantially higher response rates than placebo (46% versus 7%) and significantly reduced social and anticipatory anxiety symptoms.^29^ These findings provided initial support for serotonergic modulation in SAD and suggested that SSRIs could replicate the efficacy previously observed with MAOIs.

Subsequent larger, multicenter trials established the generalizability and clinical relevance of these effects. In a 12-week randomized controlled trial of approximately 90 patients, fluvoxamine was associated with significantly higher response rates than placebo (43% versus 23%) and produced improvements across symptom severity and psychosocial functioning.^30^ Importantly, benefits were observed across multiple scales, supporting both symptomatic and functional improvement and reinforcing fluvoxamine as an effective treatment for generalized SAD.

Later studies extended these findings by examining formulation and time course of response. In a large, randomized trial of controlled-release (CR) fluvoxamine (N = 300), fluvoxamine CR was superior to placebo across primary and secondary outcomes, with significant reductions in Liebowitz Social Anxiety Scale (LSAS) scores (by week 4).^31^ Furthermore, treatment was not associated with clinically significant sexual dysfunction or weight gain. Additionally, in a 12- to 24-week extension phase of a randomized trial, continued fluvoxamine CR was associated with sustained improvements compared with placebo, with ongoing, albeit attenuated, gains over time.^32^

Taken together, these studies demonstrate that fluvoxamine is an effective treatment for SAD, with evidence supporting acute efficacy, early onset of benefit, and maintenance of response.

### Fluoxetine

The SSRI fluoxetine, with a long elimination half-life and an active metabolite, norfluoxetine, which together contribute to sustained serotonergic activity and relatively stable plasma concentrations, has nonlinear pharmacokinetics at higher doses and has modest downstream effects on norepinephrine and dopamine systems. Interestingly, evidence for fluoxetine in SAD has been mixed and less robust than for other SSRIs. In one of the larger randomized trials, Davidson and colleagues compared fluoxetine, cognitive behavioral therapy, their combination, and placebo over 14 weeks.^33^ All active treatments were superior to placebo, with response rates of approximately 51% for fluoxetine and 54% for combined treatment.^33^ Notably, combined treatment did not confer additional benefit over either modality alone, and a substantial proportion of patients remained symptomatic, highlighting limitations of both pharmacologic and psychotherapeutic approaches. By contrast, smaller placebo-controlled trials of fluoxetine failed to demonstrate clear efficacy. In a 14-week randomized study of 60 patients, fluoxetine did not separate from placebo despite improvement in both groups.^34^

Comparative data further contextualize these findings. In a randomized trial comparing cognitive therapy with fluoxetine plus self-exposure, cognitive therapy was superior at post-treatment and follow-up, while fluoxetine did not differ from placebo on primary social anxiety outcomes.^35^ These results suggest that, while fluoxetine may provide some benefit, its effects in SAD are less consistent and may be more modest relative to both psychotherapy and other SSRIs.

### Paroxetine

Paroxetine was among the first SSRIs systematically evaluated for the treatment of SAD, and the extant evidence spans early proof-of-concept studies, randomized placebo-controlled trials, and formulation-specific investigations.^36^^,^^37^ Collectively, these studies helped establish SSRIs as first-line pharmacologic treatments for SAD and provide insights into treatment response, relapse risk, and dosing strategies.

Initial evidence came from a small open-label trial followed by a randomized discontinuation study. In this investigation, 36 adults with generalized social phobia received forced-titration of open-label paroxetine for 11 weeks, reaching a mean dose of approximately 48 mg/day.^38^ Among the 30 completers, 77% were classified as responders based on a Clinical Global Impression–Improvement (CGI-I)^39^ score of “much improved” or “very much improved.” Symptoms decreased substantially on the Liebowitz Social Anxiety Scale (LSAS).^40^ In this trial, responders were then randomized to continued paroxetine or placebo for 12 weeks; relapse occurred in 1 of 8 patients continuing paroxetine compared with 5 of 8 switched to placebo, suggesting that early discontinuation may be associated with high relapse risk.^38^

Subsequently in a multicenter, double-blind study of 290 adults treated for 12 weeks with flexibly-dosed paroxetine (20–50 mg/day) or placebo, paroxetine produced significantly greater reductions in LSAS scores compared with placebo (−29.4 versus −15.6; *P* ≤ 0.001) and higher responder rates on the CGI-I (65.7% versus 32.4%; *P* < 0.001).^41^ In this trial, differences between groups emerged by week 4, and paroxetine was generally well tolerated.^41^

Similarly, Stein and colleagues conducted a randomized placebo-controlled trial involving 187 adults with generalized social phobia. Following a placebo run-in period, participants received paroxetine (20–50 mg/day flexible dosing) or placebo for 11 weeks. At endpoint, 55% of patients receiving paroxetine were rated as “much improved” or “very much improved” compared with 24% receiving placebo (odds ratio 3.88).^42^ LSAS scores declined by 39% in paroxetine-treated patients compared with 17% of those receiving placebo.

International data have been generally consistent. In a randomized double-blind placebo-controlled study conducted across nine sites in South Africa (*N* = 93), flexibly dosed paroxetine was superior to placebo on both improvement in LSAS score and CGI-I responder rates, while adverse event rates were comparable between groups.^43^

Subsequent work examined dosing strategies. In a large fixed-dose trial involving 384 adults with generalized SAD, participants were randomized to paroxetine 20 mg/day, 40 mg/day, 60 mg/day, or placebo for 12 weeks. Improvements in LSAS scores were significantly greater with 20 mg/day compared with placebo (*P* < 0.001), while responder rates were significantly higher with 40 mg/day (*P* = 0.012).^44^

Subsequent investigations expanded the evidence base for paroxetine by examining its effects in populations with clinically relevant psychiatric comorbidity. Because early SSRI trials frequently excluded individuals with alcohol use disorders, Book and colleagues conducted a 16-week double-blind, placebo-controlled trial in 42 adults with SAD and co-occurring alcohol use disorder.^45^ In this study, paroxetine produced significantly greater reductions in social anxiety symptoms compared with placebo, as measured by both the total and subscale scores of the LSAS and other measures of social anxiety. Importantly, these findings provided some of the first controlled evidence supporting SSRI treatment of social anxiety in patients with comorbid alcohol use disorders, a population frequently encountered in clinical practice.^45^

Paroxetine has also been evaluated in active-comparator trials.^46^ In a large multicenter randomized study of 440 adults with generalized SAD, flexibly-dosed venlafaxine extended-release (ER) (75–225 mg/day), paroxetine (20–50 mg/day), or placebo was administered for up to 12 weeks. Both active treatments were significantly superior to placebo across multiple outcomes, including LSAS scores and responder rates based on CGI-I criteria. Week 12 response rates were 62.5% for paroxetine and 58.6% for venlafaxine ER compared with 36.1% for placebo (*P* < 0.001 for both comparisons). Although venlafaxine ER demonstrated earlier separation from placebo on some measures, overall efficacy and tolerability were comparable between the two antidepressants.^46^

Pharmacologic augmentation strategies have also been explored in patients with incomplete response to paroxetine.^47^ In a double-blind, placebo-controlled crossover study, Stein and colleagues evaluated whether the β_1/2_-adrenergic antagonist and 5-HT_1A_ partial agonist, pindolol, could potentiate the effects of paroxetine in patients with generalized social phobia who had not achieved a robust response after ≥ 10 weeks of maximally tolerated doses of paroxetine. Fourteen patients received adjunctive pindolol (5 mg TID) or placebo for 4 weeks in a crossover design while continuing paroxetine. Pindolol augmentation did not produce greater improvement in social anxiety symptoms compared to placebo (LSAS and Social Phobia Inventory scores). Moreover, no participants were classified as responders during pindolol treatment.^47^

More recently, paroxetine has been evaluated in comparison with cognitive therapy and combined treatment approaches. In a randomized trial of 102 patients with SAD, of whom more than half had avoidant personality disorder, participants were assigned to paroxetine, cognitive therapy, combined cognitive therapy plus paroxetine, or pill placebo for 26 weeks. Cognitive therapy produced greater improvement than paroxetine alone and placebo but was not superior to the combination treatment. At 12-month follow-up, the cognitive therapy group maintained significantly greater benefits than both paroxetine alone and placebo, whereas combined treatment did not confer an advantage over monotherapy. Recovery rates at follow-up were 68% for cognitive therapy, 40% for combination treatment, 24% for paroxetine, and 4% for placebo. These findings suggest that while paroxetine is effective for reducing symptoms of SAD, cognitive therapy may yield more durable benefits, and combining pharmacotherapy with cognitive therapy does not necessarily enhance long-term outcomes.^48^

Controlled-release formulations were evaluated in a double-blind study of 370 adults with SAD randomized to paroxetine CR (12.5–37.5 mg/day) or placebo for 12 weeks. Paroxetine CR produced significantly greater improvement in LSAS scores (difference − 13.33; *P* < 0.001) and higher responder rates on the CGI-I (57% versus 30%; odds ratio 3.12), with comparable discontinuation rates due to adverse events.^49^

Taken together, these studies suggest that paroxetine is an effective and generally well-tolerated pharmacologic treatment for adults with SAD. Across trials, paroxetine consistently produced greater improvements in LSAS scores and higher response rates relative to placebo, with separation typically emerging within the first month of treatment. These findings helped establish SSRIs as foundational treatments for SAD and informed subsequent investigations of other serotonergic antidepressants in this disorder.

The side effect profile of paroxetine is relatively unique among SSRIs. In this regard, paroxetine blocks both serotonin and norepinephrine reuptake and has significant muscarinic cholinergic receptor antagonism and may have some antihistamine effects as well. Additionally, paroxetine undergoes significant first-pass metabolism, is highly protein-bound, and is extensively metabolized by *CYP2D6*, which is encoded by a highly polymorphic gene. Moreover, paroxetine has a short half-life, and its variability at steady-state plasma concentrations may be further affected by *CYP2D6* activity/phenotype^50^ in addition to paroxetine’s binding to the CYP2D6 enzyme.^51^ Because of its short half-life, SSRI withdrawal symptoms may be more common in paroxetine-treated patients relative to those treated with other SSRIs.^52^^–^^54^

### Sertraline

The SSRI sertraline is a potent inhibitor of the serotonin transporter and, relative to other SSRIs, has modest dopamine transporter (DAT) inhibition and σ _1_ receptor binding. Sertraline is slowly absorbed from the gastrointestinal tract, with peak serum concentrations typically occurring 6–8 hours after administration.^55^ Absorption may be modestly accelerated by food intake. Sertraline is primarily metabolized through CYP2C19 and CYP2B6 pathways and has an elimination half-life of approximately 26–32 hours in adults, although interindividual variability in drug exposure has been observed, in part related to CYP2C19 and CYP2B6 phenotype.^55^

Early placebo-controlled evidence was provided by a small double-blind crossover study in which 12 patients with social phobia received sertraline (50–200 mg/day) and placebo for 10 weeks each. Treatment with sertraline resulted in significantly greater reductions in LSAS scores compared with placebo.^56^ Later, larger randomized trials confirmed these findings. In a multicenter double-blind study, Van Ameringen and colleagues randomized 204 adults with generalized social phobia to flexibly-dosed sertraline (50–200 mg/day) or placebo for 20 weeks. Sertraline-treated patients had significantly higher response rates on the CGI scores (53% versus 29%), and greater reductions in social anxiety symptoms across multiple scales, and sertraline was generally well tolerated.^57^

Similarly, a large randomized placebo-controlled trial of sertraline in patients with severe generalized SAD demonstrated significant improvements in both symptom severity and responder rates. In this study, more than 400 participants received sertraline (50–200 mg/day) or placebo for 12 weeks. Sertraline produced greater reductions in LSAS scores compared to placebo (−31.0 versus −21.7, *P* = 0.001) and significantly higher CGI-I responder rates (approximately 47% versus 26%, *P* < .001).^58^

The durability of sertraline response has also been examined. In a relapse-prevention study, patients who had responded to 20 weeks of sertraline were randomized to continue sertraline or to switch to placebo for an additional 24 weeks. Relapse occurred in only 4% of patients continuing sertraline compared with 36% of those switched to placebo.^59^

Sertraline has also been evaluated in combination with behavioral interventions. Additional randomized data further support a role for sertraline in increasing the acquisition of social skills during psychotherapy. In a 20-week double-blind trial, patients with SAD were randomized to sertraline (50–200 mg/day) or placebo in combination with either group CBT or group psychodynamic therapy. Across conditions, sertraline was associated with greater improvement in social anxiety symptoms compared with placebo, and combined treatment (sertraline plus psychotherapy) was superior to psychotherapy alone. Notably, sertraline potentiated group CBT by enhancing social skills acquisition, as reflected by greater improvements on measures of social expression, although differences between psychotherapy modalities were not observed.^60^ These findings suggest that, beyond reducing symptom severity, SSRIs may facilitate engagement with and benefit from skills-based interventions.^60^ Additionally, in a large randomized general-practice trial involving 387 patients, participants received sertraline (50–150 mg/day) or placebo for 24 weeks and were additionally randomized to exposure therapy or usual care. Sertraline treatment significantly improved social anxiety symptoms compared with placebo, and combined treatment with sertraline plus exposure therapy produced the greatest overall improvement (Blomhoff et al.^59^).

Long-term follow-up from this cohort suggested that the durability of treatment effects may differ across modalities. At one-year follow-up, patients receiving exposure therapy alone continued to improve, whereas those treated with sertraline—with or without exposure—demonstrated some deterioration after medication discontinuation, highlighting the potential importance of continued pharmacotherapy or sustained behavioral treatment.^61^

Finally, sertraline has been compared with short-term dynamic psychotherapy, and a waiting-list control group, both active treatments significantly reduced symptoms of social anxiety relative to no treatment, with no significant differences between psychotherapy and pharmacotherapy groups.^62^

Taken together, these studies demonstrate that sertraline is an effective and generally well-tolerated pharmacologic treatment for SAD. Across trials, sertraline consistently produced greater improvements in social anxiety symptoms and higher response rates relative to placebo, with evidence also supporting its role in relapse prevention and its use alongside behavioral interventions.

## Serotonin-norepinephrine reuptake inhibitors

### Duloxetine

Duloxetine is readily absorbed following oral administration and, across clinically relevant doses, produces similar inhibition of serotonin and norepinephrine transporters, without the marked dose-dependent shift toward noradrenergic activity observed with venlafaxine. Duloxetine is FDA-approved for GAD, major depressive disorder, and several pain-related conditions. While it has been extensively studied in GAD across children, adolescents,^63^ adults^64^ and older adults,^65^ evidence in SAD is limited.

The extant data are primarily derived from a small randomized, prospective study examining both initial response and dose optimization. In this trial, 39 patients with generalized SAD received open-label duloxetine (60 mg/day) for 6 weeks, with improvement in social anxiety symptoms. Patients who remained symptomatic were then randomized to continue 60 mg/day or increase to 120 mg/day for an additional 18 weeks. Although symptoms continued to improve over time, titration did significantly reduce symptoms (at a 5% threshold) although there was a moderate effect size (Cohen’s d = 0.57). Additionally, remission rates were modest, and discontinuation rates were relatively high.^66^ Taken together, these findings suggest that duloxetine may have some efficacy in SAD, but its effects appear less robust and less well established than those observed in GAD or with other first-line agents.

### Venlafaxine

Venlafaxine, an SNRI, has been FDA-approved for GAD, major depressive disorder, panic disorder, and SAD. Its active metabolite, O-desmethylvenlafaxine, contributes to its clinical effects, with relatively greater noradrenergic activity at higher exposures. However, venlafaxine is metabolized by CYP2D6, and interindividual variability in metabolism may influence both efficacy and tolerability, particularly with regard to the relative ratio of venlafaxine to O-desmethylvenlafaxine, with the latter having more noradrenergic activity.^67^

Evidence for venlafaxine in SAD is based on several large, randomized controlled trials demonstrating efficacy comparable to SSRIs. In a multicenter trial of over 400 patients, venlafaxine extended-release (75–225 mg/day) was superior to placebo across symptom severity and response outcomes, with effects emerging early in treatment and comparable efficacy to paroxetine.^68^ Similarly, a second large randomized trial confirmed significant improvements in social anxiety symptoms and response rates with venlafaxine, again demonstrating outcomes comparable to paroxetine and superior to placebo.^46^ While these trials support venlafaxine as an effective treatment for SAD, with efficacy broadly similar to first-line SSRIs. However, a recent meta-analysis failed to demonstrate superiority over placebo.^69^

From a clinical perspective, medication tolerability and treatment discontinuation effects are important considerations. Venlafaxine is associated with dose-related adverse effects, including nausea, insomnia, irritability, and increases in blood pressure, as well as sexual dysfunction and autonomic symptoms. Notably, discontinuation symptoms with venlafaxine are among the most prominent of any antidepressant and may complicate both adherence and medication transitions.^54^ Thus, while venlafaxine may represent a reasonable alternative to SSRIs in SAD, particularly in patients with partial response to serotonergic agents, its use may be limited by tolerability concerns and medication discontinuation effects, and its relative advantage over SSRIs remains uncertain.

## Atypical antidepressants

### Vilazodone

Vilazodone combines inhibition of the serotonin transporter with partial agonism at the 5-HT_1A_ receptor, and this dual mechanism may enhance serotonergic neurotransmission while modulating receptor signaling (i.e., similar to SSRI + 5-HT_1A_ agonist). Vilazodone is approved by the U.S. Food and Drug Administration for the treatment of MDD in adults.

In a 12-week double-blind, placebo-controlled trial, Careri and colleagues randomized 44 adults with generalized SAD to flexibly-dosed vilazodone or placebo. The primary outcome was change in the LSAS. At study endpoint, vilazodone-treated patients demonstrated significantly greater improvement in LSAS scores compared with placebo (mean difference approximately 14 points; Cohen’s d: 0.58). Participants entered the trial with marked to severe illness severity, and the medication was generally well tolerated.^70^

Although these findings suggest that vilazodone may have anxiolytic effects in SAD, the small sample size precludes firm conclusions regarding its efficacy. As with other newer serotonergic antidepressants evaluated in SAD, additional controlled studies are needed before vilazodone can be considered an established pharmacologic treatment for this disorder.

### Mirtazapine

Mirtazapine acts primarily through antagonism of presynaptic α₂-adrenergic autoreceptors and heteroreceptors, resulting in increased release of norepinephrine and serotonin. It is also an antagonist at several serotonin receptors, including 5-HT_2A_, 5-HT_2C_, and 5-HT_3_ receptors, while sparing 5-HT_1A_–mediated transmission. In addition, it is a potent histamine H_1_ receptor antagonist. This pharmacologic profile may confer anxiolytic effects while ostensibly reducing certain serotonergic adverse effects associated with SSRIs. Evidence supporting the use of mirtazapine in SAD is limited but suggests potential benefit. In an early open-label pilot study, adults with generalized SAD (*N* = 14) received mirtazapine (30 mg/day) for 12 weeks. Among the 12 patients who completed the study, five (42%) were classified as responders based on Clinical Global Impression-Improvement ratings and reductions in LSAS scores. Mirtazapine was generally well tolerated, although two patients discontinued treatment because of adverse effects.^71^

Subsequent randomized controlled data are sparse but provide some support for efficacy. In a double-blind placebo-controlled trial involving 66 women with social phobia, participants were randomized to receive mirtazapine or placebo for 10 weeks. Mirtazapine-treated patients had significantly greater improvements in Social Phobia Inventory and LSAS scores compared with placebo and improved health-related quality of life.^72^

However, in a randomized double-blind placebo-controlled trial, Schutters and colleagues assigned 60 patients with generalized SAD to receive mirtazapine (30–45 mg/day) or placebo for 12 weeks.^73^ The study found no significant differences between mirtazapine and placebo on changes in LSAS scores or responder rates, with approximately 13% of patients in each group meeting response criteria. These findings highlight the inconsistent evidence base for mirtazapine in SAD.^73^

Taken together, these studies suggest that mirtazapine may have anxiolytic effects in adults with SAD, although the available evidence base is small relative to that for SSRIs and SNRIs. Consequently, mirtazapine is not typically considered a first-line pharmacologic treatment for SAD but may represent an alternative in selected patients, particularly when sedation or appetite stimulation is clinically desirable or when first-line serotonergic antidepressants are poorly tolerated.

### Vortioxetine

Vortioxetine is a multimodal serotonergic antidepressant that combines inhibition of the serotonin transporter with direct modulation of several serotonin receptors, including agonism at 5-HT_1A_ receptors and antagonism at 5-HT_1D_ and 5-HT₇ receptors, among others. This pharmacologic profile has been proposed to enhance serotonergic neurotransmission while modulating downstream neural circuits involved in mood and anxiety regulation.

Evidence regarding vortioxetine in SAD is limited. In a 12-week double-blind placebo-controlled study, Liebowitz and colleagues evaluated vortioxetine (10–20 mg/day) in patients with MDD and comorbid SAD. The primary outcome was a composite CGI-I responder rate reflecting improvement in both depressive and social anxiety symptoms. Although the difference between vortioxetine and placebo did not reach statistical significance on the primary endpoint (50% versus 30% responders), vortioxetine-treated patients had significantly greater improvements on dimensional measures of both depression and social anxiety (i.e., Montgomery–Åsberg Depression Rating Scale and LSAS). Improvements in depressive symptoms tended to precede reductions in social anxiety symptoms, and the medication was generally well tolerated.^74^

Taken together, these findings suggest that vortioxetine may have benefit for patients with SAD with comorbid depressive disorders. However, given the small sample size and the absence of trials specifically targeting primary SAD, evidence supporting vortioxetine as a treatment for SAD remains preliminary.

### Benzodiazepines

Benzodiazepines bind to the γ-aminobutyric acid A (GABA_A_) receptor and function as positive allosteric modulators, potentiating the inhibitory effects of endogenous GABA. Prior to the introduction of selective SSRIs and SNRIs, benzodiazepines represented the primary pharmacologic treatment for anxiety disorders. These agents vary in duration of action, lipophilicity, abuse liability, and metabolism,^75^ but all produce rapid anxiolytic effects, often emerging shortly after administration.^75^^,^^76^

Although benzodiazepines have been studied extensively in generalized anxiety disorder and panic disorder, fewer trials have examined their efficacy specifically in SAD.^76^ In a double-blind, placebo-controlled trial of outpatients with social phobia (N = 75), clonazepam (mean maximum dose 2.4 mg/day) produced greater improvement than placebo across multiple symptom domains, including fear and avoidance, interpersonal sensitivity, and disability measures, with treatment differences evident as early as the first week of treatment.^77^ Similarly, in a randomized trial comparing clonazepam with cognitive-behavioral group therapy for social phobia, both interventions produced substantial improvement over 12 weeks, with clonazepam demonstrating somewhat greater improvement on several clinician-rated measures at study endpoint.^78^

These findings are consistent with meta-analytic evidence demonstrating that benzodiazepines produce more rapid improvement in anxiety symptoms than SSRIs or SNRIs,^79^ with measurable benefits emerging within the first week of treatment, although by approximately 8 weeks outcomes are generally comparable across medication classes.

Despite this rapid onset, the use of benzodiazepines in SAD is often limited by tolerability, misuse, and dependence concerns.^80^^,^^81^ Current prescribing guidelines generally recommend restricting benzodiazepines in adults to short-term treatment (3–6 months),^80^ although this time frame may not address the chronic course of anxiety disorders. Nevertheless, some have noted that benzodiazepines may represent a reasonable option for selected patients—particularly those at low risk for substance misuse—when other treatments are contraindicated or ineffective, provided that treatment occurs with close monitoring.^81^

In clinical trials and observational studies, the most common adverse events associated with benzodiazepines include somnolence, fatigue, dizziness or unsteadiness, which are generally dose-dependent and often emerge early in treatment. Benzodiazepines have also been evaluated as adjunctive treatments during initiation of antidepressant therapy, particularly in patients who experience transient increases in anxiety when starting SSRIs, although this has not been examined in patients with SAD specifically. For example, in adults with panic disorder initiating sertraline, adjunctive clonazepam was associated with more rapid symptom stabilization compared with placebo.^82^ Similarly, in a randomized trial of adults with generalized SAD treated with paroxetine, response rates were higher among patients receiving combined paroxetine and clonazepam compared with paroxetine and placebo (79% vs 43%), although the difference was only statistically significant at the 6% threshold (*P* = 0.06).^83^

Taken together, these findings suggest that while benzodiazepines can provide rapid symptomatic relief in SAD, their long-term role is limited by regulatory, safety, and tolerability considerations. Accordingly, they are most often used as short-term treatments or adjunctive strategies, rather than as first-line monotherapy.

## Tricyclic antidepressants

The tricyclic antidepressants (TCAs) were first developed in the 1950s as antidepressants and were the mainstay of psychopharmacotherapy prior to the introduction of the SSRIs in the late 1980s. The TCAs are categorized into tertiary amines (e.g., amitriptyline, clomipramine, doxepin, imipramine, and trimipramine) and secondary amines (e.g., desipramine, nortriptyline, protriptyline). Secondary amines are more effective in blocking the norepinephrine transporter, whereas the tertiary amines are more potent inhibitors of 5-HT reuptake. The TCAs are largely absorbed in the small intestine, which occurs relatively rapidly, with peak concentrations achieved within 2–4 hours. Additionally, like the SSRIs and SNRIs other than venlafaxine, the TCAs are highly protein-bound and are metabolized through the cytochrome system.^84^

While the use of TCAs has largely been supplanted by the use of SSRIs for the treatment of SAD, they still represent a viable option for patients who fail to respond after multiple trials of SSRIs, SNRIs, and adjunctive psychopharmacologic interventions. Mechanistically, the TCAs bind to norepinephrine and 5-HT transporters and increase synaptic concentrations of these biogenic amines, although they have some affinity for histaminergic, cholinergic, and α_1_ adrenergic receptors which contribute to the common side effects of dry mouth, constipation, and weight gain.^85^ Additionally, QTc prolongation with TCAs is more pronounced compared to SSRIs (7.1 msec; 95% CI 3.84–10.27)^86^ and TCAs may be fatal in overdose, largely secondary to TCA-related ventricular arrhythmia and seizures.

Early investigations of TCAs in SAD are methodologically heterogeneous. One of the earliest comparative studies of clomipramine in patients with social phobia within a cohort of phobic disorders (sample size 30–40 patients) involved clomipramine (75 to 150 mg/day). Some improvement in social anxiety symptoms was observed; however, the magnitude of effect was modest and less consistent than that reported in studies of other agents.^87^ Moreover, treatment response appeared gradual and variable, with a subset of patients demonstrating minimal or no improvement.^87^

Subsequent work by Simpson et al.^88^ examined imipramine in a prospective open-label trial of 15 patients with social phobia. Imipramine was flexibly dosed, typically in the range of 150–300 mg/day over approximately 8 weeks of treatment. While reductions in social anxiety symptoms were observed, the magnitude of improvement was modest, with relatively low response rates and a notable proportion of patients discontinuing treatment due to adverse effects. Importantly, the trajectory of response differed from that observed with more effective treatments, with slower and less pronounced symptom reduction and an absence of clear early separation or sustained improvement.

## Monoamine oxidase inhibitors

Monoamine oxidase inhibitors (MAOIs) inhibit the mitochondrial enzyme monoamine oxidase, which metabolizes and inactivates monoamine neurotransmitters including 5-HT, norepinephrine, and dopamine. Two isoforms of monoamine oxidase exist: MAO-A, which is primarily expressed in the gastrointestinal tract and central nervous system and preferentially metabolizes serotonin and norepinephrine, and MAO-B, which is found predominantly in platelets and dopaminergic brain regions. In the United States, the nonselective MAOIs phenelzine and tranylcypromine are most commonly prescribed, while the MAO-B inhibitor selegiline is also available. These agents differ in pharmacologic profiles and adverse effect burden but share the potential for clinically meaningful efficacy in anxiety disorders, including SAD.

Early clinical observations suggested that MAOIs may be particularly effective in patients with more severe or generalized forms of SAD. Several early trials, of which many involved phenelzine, demonstrated large reductions in core social anxiety symptoms and avoidance. Of note, phenelzine is a nonselective, irreversible MAOI that inhibits both MAO-A and MAO-B; however, it is also metabolized to phenethylidenehydrazine, which inhibits *GABA transaminase* (GABA-T). Thus, phenelzine also increases central GABA concentrations^89^ and this may contribute to its anxiolytic effects.

Taken together, the combination of irreversible monoamine oxidase inhibition and potential augmentation of GABAergic tone may explain why phenelzine has historically been viewed as a particularly effective MAOI for SAD. One of the earliest placebo-controlled trials of phenelzine for phobic anxiety demonstrated significant improvement relative to placebo after approximately 2 months of treatment, suggesting that the medication’s therapeutic effects may emerge gradually and require sustained dosing.^90^ Subsequent randomized trials in the 1980s and early 1990s provided more definitive evidence of phenelzine’s efficacy in SAD. In a placebo-controlled study comparing phenelzine, the beta-adrenergic antagonist atenolol, and placebo, Liebowitz and colleagues found that phenelzine produced significantly greater improvements in social anxiety symptoms after 8 weeks of treatment, whereas atenolol did not differ from placebo. Response rates in a later and larger follow-up trial were approximately 64% for phenelzine compared with 30% for atenolol and 23% for placebo, supporting the responsiveness of generalized social phobia to MAOI therapy.^91^^,^^92^

Additional data suggest that other nonselective MAOIs, including tranylcypromine, produce dose-dependent effects on social anxiety symptoms. In a 12-week double-blind trial of patients with panic disorder and comorbid SAD, tranylcypromine (60 mg/day) produced significantly greater reductions in social anxiety compared with 30 mg/day, with approximately two-fold greater improvement in LSAS scores.^93^ Interestingly, both doses reduced panic symptoms, suggesting a differential dose–response relationship across symptom types. These findings, although in a comorbid population, support the efficacy of tranylcypromine in SAD and underscore the importance of adequate dosing when targeting social anxiety.

Given concerns regarding dietary restrictions and the risk of hypertensive crises associated with irreversible MAOIs, investigators also examined reversible inhibitors of monoamine oxidase-A as potentially safer alternatives. Moclobemide, a reversible MAO-A inhibitor, was evaluated in a multicenter, double-blind, placebo-controlled dose–response trial in which patients with SAD were randomized to placebo or fixed doses of moclobemide ranging from 75 mg to 900 mg daily for 12 weeks. Although a trend toward greater improvement with higher doses was observed at 8 weeks, the study did not demonstrate significant differences between moclobemide and placebo at week 12. At endpoint, approximately 35% of patients receiving the highest dose (900 mg/day) were rated as at least “much improved,” compared with 33% of those receiving placebo. Moclobemide was generally well tolerated, with insomnia representing the only dose-related adverse event.^94^

Comparative trials have also examined the relative efficacy of pharmacotherapy and psychotherapy. In a randomized study comparing cognitive behavioral group therapy (CBGT) with phenelzine treatment, both interventions produced substantial reductions in social anxiety symptoms and were superior to control conditions. Phenelzine demonstrated earlier symptom improvement and was superior to CBGT on some outcome measures at 12 weeks, although both treatments yielded improvement.^95^

More recent work has examined whether combining pharmacotherapy and psychotherapy provides additional benefit. In a randomized placebo-controlled trial involving 128 individuals with SAD, patients were assigned to phenelzine, cognitive behavioral group therapy, combined treatment, or placebo. Both phenelzine and CBGT monotherapy produced greater reductions in Liebowitz Social Anxiety Scale scores than placebo, while the combination of phenelzine and CBGT yielded the largest overall improvements and the highest response and remission rates. At 12 weeks, response rates were 54% with phenelzine alone and 72% with combined treatment, suggesting that integration of pharmacologic and behavioral interventions enhances outcomes for some patients.^96^

Earlier work had also explored the interaction between pharmacotherapy and behavioral exposure. In a small, randomized trial of patients with agoraphobic and social phobia symptoms, exposure therapy produced significant improvement regardless of medication assignment, whereas phenelzine appeared to primarily reduce subjective anxiety during exposure rather than directly augment antiphobic effects.^97^ These findings foreshadowed later work emphasizing the complementary roles of pharmacologic and behavioral interventions.

Overall, these studies demonstrate that MAOIs, particularly phenelzine, are effective pharmacologic treatments for SAD and were among the earliest medications shown to produce robust improvements in social anxiety symptoms. Despite their efficacy, the use of MAOIs has declined substantially with the introduction of SSRIs and SNRIs, largely due to dietary restrictions, drug–drug interaction risks, and tolerability concerns. Consequently, MAOIs are now generally reserved for patients with treatment-resistant SAD or those who fail to respond adequately to first-line pharmacotherapies.

## Azapirones

### Buspirone

Buspirone, a partial agonist at the serotonin 5-HT_1A_ receptor, is approved for the treatment of generalized anxiety disorder in adults. However, there are limited data in SAD. Van Vliet and colleagues randomized 30 adults with *DSM-IV* social phobia to receive buspirone (30 mg/day) or placebo for 12 weeks. No significant differences were observed between buspirone and placebo on any outcome measure, although the medication was well tolerated.^98^

## α2δ calcium channel ligands

### Gabapentin

Gabapentin, an analogue that modulates voltage-gated calcium channels via the α2δ subunit, has been evaluated in SAD in a single randomized controlled trial. In this 14-week, double-blind study (N = 69), flexibly-dosed gabapentin (900–3600 mg/day) was associated with significantly greater reductions in social anxiety symptoms compared with placebo across clinician- and patient-rated measures.^99^ In this study, gabapentin was generally well tolerated, with adverse effects including dizziness, somnolence, and gastrointestinal symptoms, consistent with its known profile. However, despite these positive findings, the evidence base remains limited, with no replication studies or clear dose–response data.^99^ Taken together, gabapentin may represent a reasonable off-label option in selected patients, particularly when traditional agents are not tolerated, but its role in SAD remains unclear.

### Pregabalin

Pregabalin is a γ-aminobutyric acid (GABA) analogue that binds with high affinity to the α₂δ subunit of voltage-gated calcium channels, thereby reducing presynaptic calcium influx and decreasing calcium-dependent neurotransmitter release.^100^ Unlike benzodiazepines, pregabalin does not bind directly to GABA_A_ receptors, enhance GABA currents, or affect GABA metabolism, nor does it demonstrate functional activity at serotonin, dopamine, or norepinephrine receptors. Pregabalin is rapidly absorbed from the gastrointestinal tract, has high bioavailability, and is eliminated primarily through renal clearance.

Pregabalin has been evaluated in several randomized controlled trials in adults with generalized SAD. In a 10-week multicenter placebo-controlled trial, 135 patients were randomized to pregabalin 150 mg/day, 600 mg/day, or placebo. Treatment with pregabalin 600 mg/day was associated with a significantly greater reduction in LSAS scores compared with placebo, whereas the 150 mg/day dose did not differ from placebo.^101^ Additional fixed-dose studies similarly demonstrated that 600 mg/day produced significantly greater reductions in LSAS scores compared with placebo, with improvement evident as early as week 1.^102^

Pregabalin has also been examined in relapse prevention. In a study of patients who responded to 10 weeks of open-label pregabalin (450 mg/day), continuation of pregabalin significantly delayed time to relapse compared with placebo during a 26-week double-blind phase.^103^

Across trials, pregabalin was generally well tolerated, with dizziness, weight gain and somnolence representing the most commonly reported adverse events. Collectively, these findings suggest that pregabalin (particularly at doses of 450–600 mg/day) may be an effective treatment option for adults with generalized SAD.

## GABA-related interventions

### Tiagabine

Tiagabine is a GABA reuptake inhibitor that exerts its effects through blockade of the presynaptic GAT-1 transporter, thereby increasing extracellular GABA concentrations. Interest in tiagabine as an anxiolytic arose from evidence suggesting that enhancement of GABAergic signaling may reduce anxiety symptoms. Notably, its use has been limited by safety concerns, including reports of de novo seizures in individuals without epilepsy, which led to regulatory warnings and has contributed to its declining use.

In an open-label 12-week trial, Dunlop and colleagues treated adults with SAD using tiagabine (4–16 mg/day). Among the intent-to-treat sample (*N* = 54), improvement was observed across multiple outcomes, including the LSAS and CGI. Approximately 41% of participants were classified as responders, and tiagabine was generally well tolerated.^104^ Interestingly, tiagabine increases activity in the ventromedial prefrontal cortex in generalized SAD, and this region is implicated in emotional regulation and fear processing. Moreover, baseline vmPFC activity predicted treatment response, suggesting that modulation of corticolimbic circuitry may contribute to therapeutic effects in SAD.^105^

Taken together, the limited available evidence suggests that tiagabine may have potential anxiolytic effects in SAD, but controlled clinical trials are lacking. Compared with GAD, where tiagabine has been studied more extensively, evidence supporting its efficacy in SAD remains preliminary.

## Second-generation antipsychotics

### Cariprazine

Cariprazine is a dopamine D₂/D₃ receptor partial agonist with preferential affinity for D₃ receptors, as well as activity at several serotonergic receptors including 5-HT_1A_, 5-HT_2A_, 5-HT_2B_, 5-HT_2C_, and 5-HT₇. In a 12-week study, participants with generalized SAD (*N* = 40) were randomized to cariprazine 1.5 mg/day or placebo, with the option to increase to 3 mg/day after week 8 for insufficient response.^106^ Cariprazine produced a significantly greater reduction in LSAS scores compared with placebo (difference 15.4 points, p = 0.032; Cohen’s d = 0.71), with separation from placebo emerging as early as week 1. Response rates were higher with cariprazine (65%) than placebo (40%). Additionally, cariprazine was generally well tolerated, although akathisia occurred more frequently in the active treatment group.^106^

### Olanzapine

The second-generation antipsychotic, olanzapine, a D₂ and serotonin 5-HT_2A_, H₁ and α₁-adrenergic receptor antagonist, has been evaluated in one 8-week randomized, double-blind, placebo-controlled pilot study of adults with SAD (*N* = 12). In this trial, olanzapine (5–20 mg/day) produced greater improvement than placebo on the Brief Social Phobia Scale and Social Phobia Inventory.^107^ Olanzapine was associated with drowsiness and dry mouth.

### Quetiapine

Quetiapine is an atypical antipsychotic with antagonistic activity at multiple neurotransmitter receptors, including 5-HT_2A_, D₂, H₁, and α₁-adrenergic receptors. In addition, its active metabolite, norquetiapine, is a potent norepinephrine transporter inhibitor, antagonist of 5-HT_2B,_ and partial agonist of 5-HT_1A_. Although quetiapine has moderate affinity for the D₂ receptor, it dissociates rapidly from this receptor, which contributes to its relatively low risk of extrapyramidal symptoms compared with other antipsychotics. Its anxiolytic and sedative properties are likely related to combined serotonergic, histaminergic, and noradrenergic receptor effects.

Evidence supporting quetiapine in SAD is limited. In an open-label study, Schutters et al.^108^ reported quetiapine-related improvement in social anxiety symptoms. However, controlled evidence is sparse. In a randomized, placebo-controlled trial evaluating quetiapine monotherapy (up to 400 mg/day) in adults with SAD, no statistically significant differences between quetiapine and placebo were observed on the Brief Social Phobia Scale at study endpoint or in the number of responders among those receiving placebo and those receiving quetiapine.^109^

We are unable to locate any reports of aripiprazole, risperidone, lurasidone, paliperidone, brexpiprazole, or lumateperone in treating adults with SAD.

## Hormonal therapy

Hormone-based interventions, particularly testosterone and oxytocin, have garnered interest as potential treatments for SAD given their roles in modulating social behavior and threat processing. These systems are closely linked to circuits involved in fear of negative evaluation and avoidance.

### Testosterone

Randomized controlled data on testosterone in SAD are limited, and most studies are in women^110^^,^^111^ and focus on mechanistic or adjunctive effects. Initial experimental work demonstrated that a single testosterone dose decreased gaze avoidance of the eye region (a behavioral marker of social submission) in women with SAD, suggesting modulation of core avoidance processes. However, these findings reflect laboratory effects rather than sustained clinical improvement.

Subsequent proof-of-concept work examined testosterone as an adjunct to exposure therapy. In a randomized trial of 55 women with SAD, administration of testosterone prior to an exposure session altered fear dynamics during subsequent exposure, characterized by higher peak fear followed by more rapid reduction.^110^ Despite this effect on fear processing, there was no significant improvement in self-reported anxiety symptoms. Secondary analyses suggested that individuals with greater baseline avoidance may derive greater benefit in terms of fear reduction, although these effects did not generalize to overall symptom severity or functional outcomes.

Complementary findings indicate that endogenous testosterone levels may predict response to exposure therapy,^112^^,^^113^ further supporting the role of testosterone as a modulator of therapeutic learning rather than a primary treatment. Taken together, these data suggest that testosterone may enhance aspects of exposure-based learning, particularly in individuals with high avoidance, but there is currently no evidence supporting its use as a standalone treatment for SAD or for improving global clinical outcomes.

### Oxytocin

Intranasal oxytocin is the most extensively studied neuropeptide in SAD, given its role in social cognition, affiliation, and threat processing. Despite strong mechanistic rationale, clinical findings have been mixed and often context-dependent.^114^ Early randomized work demonstrated that oxytocin, when administered prior to exposure therapy, improved positive self-evaluations during social performance tasks but did not reduce core symptoms of anxiety, maladaptive cognitions, or functional impairment.^115^ Subsequent studies have highlighted variability in response based on individual characteristics and context. For example, oxytocin has not consistently improved reward motivation in SAD and, in some cases, has been associated with impairments in social working memory among individuals with higher baseline anxiety.^116^ Although improvements in observed social behavior have been reported in structured interaction tasks, these effects are not consistently reflected in subjective experience or overall symptom change.

Neuroimaging studies provide additional insight, demonstrating that oxytocin modulates activity and connectivity within key regions implicated in SAD, including the amygdala, insula, and prefrontal cortex;^115^ although, these neurofunctional effects have not translated into consistent clinical benefit. Systematic reviews of randomized controlled trials reinforce this pattern, concluding that, despite biological plausibility, oxytocin has not demonstrated efficacy in reducing core symptoms of anxiety or depression, including in SAD.

### Summary and clinical implications

Collectively, hormone-based interventions highlight an important conceptual advance—targeting social and affective neurocircuitry beyond traditional monoaminergic systems. However, current evidence suggests that both testosterone and oxytocin function primarily as modulators of social behavior and therapeutic processes, rather than as effective standalone treatments.

At present, these approaches remain investigational. Their greatest potential may lie in augmenting behavioral interventions or targeting specific subphenotypes, rather than serving as broadly applicable pharmacologic treatments for SAD.

## Supplements

Several dietary supplements and herbal products have been proposed as treatments for anxiety disorders; however, evidence specifically in SAD is extremely limited. We were unable to identify controlled clinical trials evaluating magnesium oxide, lavender oil (Silexan), or *Hypericum perforatum* (St. John’s Wort) in adults with SAD.

Regarding omega-3 polyunsaturated fatty acids, observational data suggest a possible association with SAD. In a case–control study of untreated adults with SAD, erythrocyte concentrations of several n-3 fatty acids were significantly lower than in healthy controls and were inversely correlated with symptom severity on the LSAS.^117^ These findings support the “phospholipid hypothesis,” which proposes that alterations in membrane fatty acid composition may influence neuronal signaling and emotional regulation.^117^ However, no randomized clinical trials have evaluated omega-3 supplementation as a treatment for SAD, and the clinical relevance of these biochemical findings remains uncertain.

Finally, kava (*Piper methysticum*), has been used for centuries and, over the last several decades, was evaluated as a treatment for anxiety and in adults with GAD^118^ although its use in SAD is only supported by a single case report^119^ and there have been historical concerns related to potential hepatotoxicity.^120^

## Emerging treatments

### Pherines

Pherines represent a novel class of investigational compounds that are structurally related to neurosteroids but act locally within nasal chemosensory pathways rather than through systemic mechanisms. Pherines are agonists of receptors on nasal chemosensory neurons that rapidly activate neural circuits primarily from the olfactory bulb to the amygdala, hypothalamus, and entorhinal area/hippocampus that control behavior, memory, and autonomic nervous system function and broadcast to multiple other brain regions. These neural circuits reach the key brain areas to selectively restore “normal” function by triggering the brain’s natural mechanisms to maintain physiologic homeostasis, all with tolerability similar to that of placebo.^121^ Pherines are not intended to enter the systemic circulation or cross the blood–brain barrier; instead, their effects are mediated through olfactory–limbic neural circuits activated at the level of the nasal epithelium.^121^ As a result, these agents may modulate anxiety-related circuitry without producing measurable systemic drug exposure or typical central nervous system adverse effects associated with oral pharmacotherapy such as addiction, withdrawal symptoms, sedation, weight gain, and sexual dysfunction.

Fasedienol, a modulator of the olfactory-limbic amygdala fear/anxiety neurocircuits, is the most extensively studied compound within this class and has been evaluated as an intranasal treatment for SAD. In a randomized, double-blind, placebo-controlled clinical study that involved challenging women with generalized SAD, a single intranasal dose of fasedienol administered prior to simulated public speaking and social interaction tasks produced significantly greater reductions in subjective distress compared with placebo. Improvements were observed across challenge paradigms, and a significantly greater number of patients receiving fasedienol were rated as “much” or “very much improved” compared with placebo (75% versus 37%). The medication was generally well tolerated.^122^

More recent work has begun to clarify the biological mechanisms underlying these effects. In a randomized study of healthy volunteers, intranasal administration of fasedienol increased electrogram activity in nasal chemosensory receptor neurons and produced rapid reductions in autonomic nervous system parameters associated with anxiety, including respiratory rate, heart rate, and electrodermal activity. These changes were not observed following administration of steroidal hormones or controls.^123^

Taken together, these findings suggest that pherines represent a mechanistically distinct approach, potentially providing rapid anxiolytic effects through modulation of olfactory–limbic neurocircuitry without systemic drug exposure. Although early results are promising, larger randomized clinical trials are still ongoing.

### Nicotinic receptor agonists

Nicotinic acetylcholine receptors (nAChRs), particularly the α_7_ subtype, regulate attentional processes and stress-related neural circuits through modulation of glutamatergic and GABAergic neurotransmission. Because these receptors influence cognitive control and threat processing, α_7_-nAChR agonists have been investigated as potential rapid-acting or situational treatments for performance-related anxiety.

In a randomized, double-blind, placebo-controlled trial of adults (N = 230) with public speaking anxiety, a single oral dose of the α_7_-nAChR agonist VQW-765 (10 mg) prior to undergoing a Trier Social Stress Test produced a numerical reduction in anxiety compared to placebo, although the difference did not reach statistical significance.^124^ Interestingly, pharmacokinetic–pharmacodynamic analyses demonstrated an inverted U-shaped exposure–response relationship, with significant improvement among participants in the middle plasma concentration range. Exploratory analyses also suggested a greater treatment effect in women.

VQW-765 was well tolerated, and no major safety concerns were observed following single-dose administration.^124^ Although these findings derive from an experimental stress paradigm rather than a clinical trial in patients with SAD, they provide preliminary support for α_7_-nicotinic receptor modulation as a potential mechanism for rapid or on-demand anxiolysis in SAD. Additional trials are ongoing (clinicaltrials.gov: NCT07221578).

### Arginine vasopressin receptor antagonists

Building on chemosensory approaches that modulate olfactory–limbic circuitry, emerging strategies increasingly target downstream neuropeptide systems and circuit-specific inhibitory mechanisms involved in social threat processing. For example, NTX-1472 is a selective, brain-penetrant vasopressin 1a (V_1a_) receptor antagonist that is being evaluated in adults with SAD. Importantly, arginine vasopressin regulates social behavior and stress responsivity,^125^ with V1a receptors expressed in key limbic regions including the amygdala and bed nucleus of the stria terminalis.^126^ Antagonism of this system represents a non-monoaminergic approach to attenuating social threat processing. The ongoing Phase 2 trial is evaluating NTX-1472 over 8 weeks in adults with SAD (clinicaltrials.gov: NCT07323784).

Together, these approaches reflect a shift from global neurotransmitter modulation toward targeted engagement of neural systems underlying social anxiety, with the goal of improving both efficacy and tolerability.

## Situational pharmacologic strategies and clinical use patterns

An important distinction in the pharmacologic management of SAD relates to the heterogeneity of clinical presentation, particularly between generalized and performance-only subtypes. While randomized controlled trials largely focus on patients with generalized SAD and support the use of continuous pharmacotherapy (e.g., SSRIs and SNRIs), many commonly used real-world strategies involve PRN medication use, although these have not demonstrated consistent benefit in controlled studies.

β-adrenergic antagonists represent a prototypical example of this divergence between clinical practice and randomized controlled trial-based evidence. Although early studies failed to demonstrate the efficacy of beta-blockers in generalized SAD, these agents are frequently used in a PRN manner for performance-related anxiety. Their effects are largely peripheral, attenuating autonomic symptoms such as tachycardia, tremor, and adrenergic arousal, rather than directly modifying the cognitive or affective components of anxiety. As such, beta-blockers may be most useful in patients with circumscribed performance anxiety in whom somatic symptoms predominate.

Similarly, benzodiazepines are sometimes used on an as-needed basis in anticipation of discrete anxiety-provoking situations. While these agents can reduce subjective anxiety rapidly, their use in this context is difficult to establish in traditional RCTs. Further, benzodiazepines, while they acutely reduce anxiety, may interfere with exposure-based learning and fear extinction, potentially limiting long-term improvement.

Finally, alcohol use represents a common, though maladaptive, form of “as-needed” anxiolysis in patients with SAD.^127^ Many individuals report transient reductions in social anxiety and inhibition following drinking, likely mediated by facilitation of GABAergic signaling and attenuation of threat-related neural circuitry. However, this short-term relief comes at the cost of reinforcing avoidance-based coping and may interfere with corrective learning during social exposure. Over time, this pattern is associated with increased risk of alcohol use disorder, a well-documented comorbidity in SAD,^128^ and may ultimately worsen both anxiety severity and functional impairment. From a clinical perspective, the use of alcohol as a situational anxiolytic highlights the importance of identifying patients who rely on pharmacologic (or substance use)-based strategies to manage social anxiety, and of redirecting treatment toward interventions that produce more durable and adaptive change.

Taken together, these observations highlight a broader principle: pharmacologic strategies (whether prescribed or self-medication) that target acute physiological arousal may provide situational benefit in selected patients but do not address the core cognitive and behavioral mechanisms of SAD.

## Guidelines for the treatment of SAD

**Figure 1.**
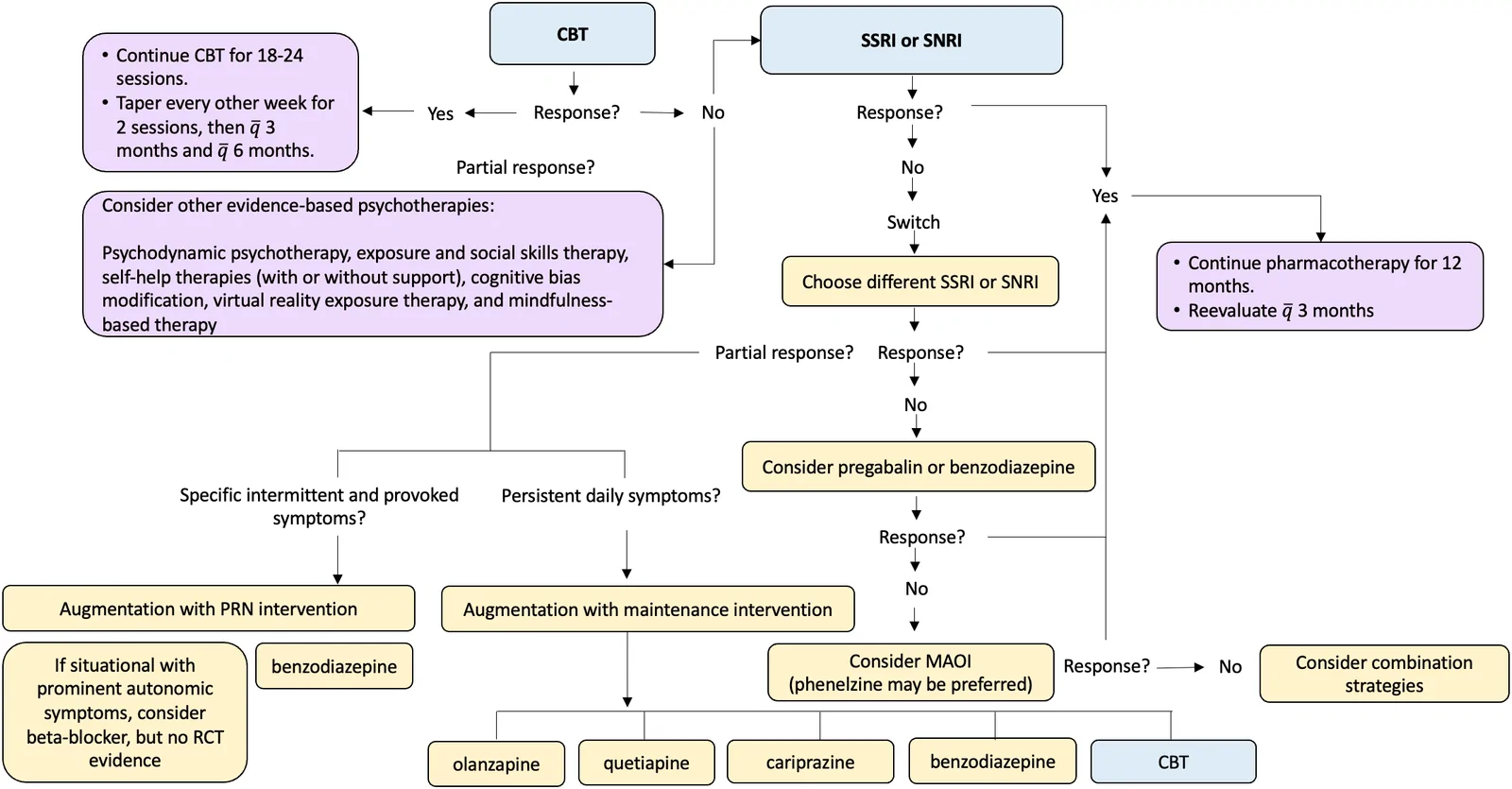
Psychopharmacologic treatment algorithm for social anxiety disorder in adults. Figure 1. long description.

The extant literature suggests that multiple pharmacologic classes are available to clinicians treating SAD. Collectively, these agents represent a pharmacologic armamentarium rather than an interchangeable list. Most treatment guidelines converge in recommending SSRIs as first-line pharmacotherapy, followed by SNRIs when response is inadequate or tolerability limits use, although some guidelines do not.^20^ Algorithm-based treatment frameworks provide a structured approach to sequencing these interventions (Figure 1). For example, the Psychopharmacology Algorithm Project at the Harvard South Shore Program recommends initial treatment with an SSRI, followed by switching to another SSRI or to an alternative agent such as venlafaxine if response is insufficient. In cases of persistent symptoms, subsequent steps may include trials of medications such as mirtazapine, clonazepam, or phenelzine, with additional strategies including augmentation approaches (e.g., buspirone). These algorithmic approaches emphasize stepwise treatment, adequate trial duration, and systematic switching or augmentation.

Ultimately, the choice of medication for the treatment of SAD depends on multiple patient-specific factors, including target symptoms, age, comorbidities, pharmacokinetic and pharmacodynamic interactions, and clinically relevant genetic polymorphisms in CYP450 genes. In this regard, the available pharmacotherapies should be viewed not as interchangeable options but as components of a broader and evolving psychopharmacologic armamentarium. This review highlights that the pharmacologic treatment of SAD, with all of its complexities, continues to evolve. Regardless of the specific intervention, whether pharmacologic, psychotherapeutic, or combined, achieving early clinical improvement and remission should remain the primary treatment goal.

## Treatment-resistant social anxiety disorder

Conceptualizing treatment resistance in SAD remains challenging, as standardized definitions for treatment resistant anxiety, in general, were only recently proposed and are inconsistent across studies. Recent Delphi-based consensus criteria suggest that pharmacologic treatment resistance in anxiety disorders should be defined by failure to achieve clinically meaningful improvement (i.e., <50% symptom reduction) following at ≥ 2 adequate trials of first-line pharmacotherapies (e.g., SSRIs or SNRIs), at therapeutic doses for ≥ 6–8 weeks with confirmed adherence (Table 1).^129^ Importantly, these definitions also emphasize the need to exclude “pseudo-resistance,” including subtherapeutic dosing, insufficient duration, poor adherence, or unaddressed comorbidity.^129^ In parallel, failure of CBT, particularly exposure-based interventions, is a complementary dimension of treatment resistance. Together, these data underscore that treatment-resistant SAD is best understood not as a binary construct, but as a spectrum reflecting incomplete response across pharmacologic and behavioral interventions.

**Table 1.** Proposed Criteria for Treatment-Resistant Anxiety Disorders and Considerations for Defining Treatment-Resistance in Social Anxiety Disorder (SAD) Table 1. long description.

	Consensus Definition (Delphi-based)^129^	Considerations in SAD
Treatment failure	Failure to achieve clinically meaningful improvement. Typically defined as <50% reduction in symptom severity or CGI-I ≤ 2	Persistent clinically significant social anxiety (e.g., avoidance, fear of negative evaluation) despite treatment Lack of functional improvement is often as important as symptom scores
Pharmacologic treatment resistance	≥2 failed trials of first-line pharmacotherapy (e.g., SSRI/SNRI), from different classes Treatment should be at adequate dose and duration (≥6–8 weeks), with adherence	Ensure true adequacy as many “failures” reflect underdosing or early discontinuation Consider switching within SSRI class before moving to alternative mechanisms
Psychotherapy treatment resistance	≥1 failed trial of adequately delivered first-line psychotherapy (e.g., CBT with exposure) Must have sufficient intensity and duration (≈12–20 weeks)	Critically evaluate exposure quality: many patients receive CBT without sufficient exposure intensity; avoidance often persists despite pharmacotherapy alone
Pseudo-resistance (must be excluded)	Nonresponse due to inadequate dose (or exposure), duration, adherence, or confounding factors (e.g., comorbidity, pharmacokinetics)	Extremely common in SAD. Low-dose SSRI trials, poor adherence, or avoidance of exposure tasks may mimic treatment resistance
Staging model (spectrum of resistance)	Stage 1: failure of ≥2 pharmacologic trials *or* ≥ 1 psychotherapy trial Stage 2: failure of both pharmacotherapy *and* psychotherapy Stage 3: failure of multiple pharmacologic and psychotherapeutic trials (multi-modal TR-AD)	Distinguishes “partial SSRI failure” from true multi-modal resistance Stage 2–3 patients often require combined or non-standard approaches
Comorbidity	Comorbidities should not define resistance but should be documented and considered post hoc	Depression, substance use, and personality traits (e.g., avoidance) may complicate SAD and contribute to apparent resistance

Despite these emerging definitions, most data informing the management of treatment-resistant SAD derive from small or uncontrolled studies, with only a limited number of randomized comparisons for next-step treatment decisions. A recent systematic review^130^ identified a small number of studies in treatment-resistant SAD, including antidepressant switching and augmentation approaches. Across these studies, switching within the SSRI class appears reasonable. For example, in an open-label trial of patients who had failed paroxetine, escitalopram produced response in approximately 48% over 12 weeks and was well tolerated, suggesting that nonresponse to one SSRI does not preclude benefit from another.^131^

Evidence for antidepressant combination strategies is limited. In patients who failed mirtazapine, the addition of paroxetine yielded response rates similar to paroxetine alone, suggesting that antidepressant combination may have limited additional benefit in terms of efficacy, although there may be modest advantages in tolerability (e.g., sexual side effects).^132^

Data for SNRIs in SSRI-resistant populations are mixed. A small open-label study suggested that venlafaxine reduces social anxiety and avoidant behavior in SSRI nonresponders^.^^133^ However, in a randomized trial of patients with persistent symptoms following an adequate trial of sertraline, switching to venlafaxine did not outperform continued sertraline, calling into question its role as a second-line strategy, at least with regard to switching to an SNRI.^133^

Monoamine oxidase inhibitors remain an important, though underutilized, option. In a small case series of previously treatment-resistant patients, phenelzine was associated with a high response rate (6/9), with some patients experiencing marked functional recovery and sustained benefit with continued treatment.^134^

Augmentation strategies have been more rigorously evaluated. In a 12-week, three-arm randomized trial of 181 patients who remained symptomatic after a 10-week trial of sertraline, augmentation with clonazepam substantially improved response rates (56% versus 36%) and produced greater reductions in symptom severity and disability compared with continued sertraline, although remission rates did not differ significantly.^135^ These findings support short-term benzodiazepine augmentation in selected patients. However, not all augmentation approaches are effective. In a placebo-controlled crossover study of patients with persistent symptoms despite SSRI treatment, pindolol did not improve social anxiety symptoms, and no patients met response criteria.^47^

As mentioned above, some second-generation antipsychotics have efficacy as monotherapy for SAD. Moreover, several second-generation antipsychotics are now FDA-approved for adjunctive use with an antidepressant for MDD, and subanalyses of these data for some reveal anti-anxiety effects. This suggests that it could also be a reasonable strategy for their use in anxiety disorders, although no data have been reported to date for their adjunctive use in SAD.

In summary, these data suggest that treatment-resistant SAD is best approached through sequential optimization, strategic switching, and selective augmentation. Within-class SSRI switching remains reasonable, evidence for SNRIs as second-line agents is mixed, and benzodiazepine augmentation may offer short-term benefit in selected patients. Importantly, MAOIs still have a unique role in refractory illness and should be considered before concluding that a patient is truly treatment-resistant.

## Shortcomings of current interventions in SAD

Despite multiple effective pharmacologic options for SAD, many of these medications have significant limitations that affect tolerability, adherence, and ultimately long-term outcomes. Most notably, a substantial proportion of patients fail to achieve full remission. Further, first-line treatments, particularly SSRIs, are associated with a constellation of adverse effects that can compromise adherence and the ability of patients to continue treatment. Sexual dysfunction, weight gain, and emotional blunting are among the most clinically relevant and often underreported by patients unless directly assessed.^54^ These effects frequently emerge over time and may decrease adherence despite initial efficacy.

Benzodiazepines, which may provide rapid and reliable relief in patients with SAD, are associated with sedation, mental slowing, and anterograde memory impairment,^136^ which are dose-dependent and may persist.^137^ Psychomotor impairment,^138^ including effects comparable to low levels of alcohol intoxication, has implications for driving safety and risk of falls, particularly in older adults. Tolerance (primarily to sedation rather than anxiolytic effects) can develop; however, despite concerns regarding dose escalation and misuse, longitudinal data in adults with anxiety disorders suggest that many patients do not exhibit progressive dose escalation over time.^139^ Nonetheless, the potential for dependence, cognitive effects, and interaction with alcohol necessitates careful patient selection and generally limits benzodiazepines to short-term or adjunctive roles. Thus, while recent perspectives suggest that the risks of benzodiazepines may be overstated in some contexts, their use should remain guided by an individualized risk–benefit assessment,^75^^,^^81^ and they may not represent a viable option for some patients with comorbid substance use, advanced age, or elevated fall risk.

Antipsychotic agents, while demonstrating efficacy in some studies of SAD,^106^^–^^109^ are associated with extrapyramidal symptoms, metabolic burden, and, in some cases, sedation, all of which limit their acceptability in this population and require ongoing monitoring. Moreover, MAOIs, which may be among the most effective pharmacologic treatments for SAD, remain underutilized due to dietary restrictions, drug–drug interactions, and tolerability concerns.^140^ In the case of MAOIs, these practical limitations often outweigh their efficacy advantages in routine clinical care.

Finally, interindividual variability in pharmacokinetics and pharmacodynamics contributes to heterogeneity in both response and tolerability. For commonly used SSRIs, including sertraline, escitalopram, citalopram, and fluvoxamine, drug exposure varies substantially as a function of CYP2C19 and CYP2D6 phenotypes.^50^^,^^55^^,^^141^^,^^142^ Additionally, variation in these enzymes may require dose adjustment and has been associated with a greater likelihood of discontinuation because of side effects in poor metabolizers and a greater likelihood of discontinuation because of lack of efficacy in faster metabolizers.^143^^,^^144^ These differences may influence both efficacy and side-effect burden and, in some patients, produce clinically important fluctuations in plasma concentrations with missed doses.^145^

Taken together, these limitations highlight the need for treatments that are not only effective, but also better tolerated, more rapid-acting, and less dependent on strict adherence.

## Long-term pharmacologic treatment in SAD

SAD is typically chronic and relapsing,^1^ and pharmacologic treatment often extends beyond the acute phase of symptom reduction. Although most randomized trials evaluate short-term outcomes (generally 8–12 weeks) in the real world, treatment often involves longer periods. Decisions regarding treatment continuation therefore depend less on fixed timelines and more on the durability of clinical response, functional recovery, and patient preference.^54^

Several medication classes (e.g., SSRIs, SNRIs, benzodiazepines, and other agents) have demonstrated efficacy in SAD. Additionally, across anxiety disorders more broadly, antidepressants and benzodiazepines produce comparable overall reductions in anxiety symptoms,^79^ although their temporal profiles differ. Benzodiazepines tend to produce more rapid symptomatic improvement,^76^^,^^79^ whereas antidepressants typically show a slower onset but sustained benefit over time.^79^

These differing trajectories can influence early treatment decisions. Rapid symptom relief may facilitate re-engagement with work, school, or social situations, whereas slower-acting treatments require a longer period before benefits become apparent. Importantly, decisions regarding continuation of pharmacotherapy are rarely based on symptom reduction alone. Functional recovery—such as returning to work or school is a better measure of stability. From this perspective, pharmacotherapy in SAD should be seen as a longitudinal treatment strategy rather than a fixed-duration intervention, although some recommend a minimum treatment of at least 12 months,^146^ and this has been demonstrated to be an evidence-based strategy in antidepressant-treated adults with GAD.^147^ Finally, for some patients, continued treatment supports sustained remission and reduces relapse risk. For others who achieve durable improvement and develop effective coping strategies, a gradual attempt at medication discontinuation may be reasonable.

### Discontinuation and deprescribing in SAD

Discontinuation of pharmacotherapy in SAD requires balancing the potential benefits of reducing medication exposure against the risks of relapse or discontinuation-related symptoms. Gradual dose reduction is generally recommended when discontinuing psychotropic medications, including benzodiazepines and SSRIs, as abrupt discontinuation may increase the likelihood of withdrawal symptoms^53^^,^^148^^–^^151^ or symptomatic worsening and makes distinguishing withdrawal symptoms from relapse difficult.^54^

Although relatively few studies have examined medication discontinuation specifically in SAD, available evidence provides some guidance. One controlled study examined patients with social phobia who had responded to 6 months of clonazepam treatment and randomized them either to continued therapy or to gradual tapering with placebo substitution. Relapse occurred more frequently among those who discontinued treatment (21%) than among those who continued therapy, although most individuals undergoing tapering maintained meaningful improvement and withdrawal symptoms were generally mild.^152^

Longer-term follow-up studies also suggest that treatment gains may persist after pharmacotherapy ends. In a two-year follow-up of individuals who had participated in a clonazepam trial for social phobia, patients initially treated with active medication continued to exhibit lower symptom severity than those who had received placebo.^153^ These findings suggest that pharmacologic treatment produces longer-term functional improvements even when medication is eventually discontinued. Taken together, these data suggest that while relapse risk must be considered when tapering medication, many patients can successfully discontinue treatment when this process is gradual and clinically supervised.

### Psychological aspects of medication discontinuation in SAD

Medication discontinuation is influenced not only by pharmacologic factors but also by psychological factors.^54^ For many, medication represents either a critical support or reminder of their anxiety disorder, and these perceptions can strongly shape attitudes toward stopping medication. Patients also differ in how they attribute their improvement, with some seeing medication as the primary driver of recovery, whereas others credit behavioral change, psychotherapy, or personal resilience.^154^ Evidence suggests that these attributional styles can influence discontinuation experiences, particularly with benzodiazepines. For example, in studies examining benzodiazepine tapering, individuals who attributed improvement primarily to medication were more likely to experience distress and symptom worsening during tapering than those who attributed improvement to their own coping.^154^

Clinicians and family members may also influence discontinuation decisions.^54^ Clinicians may hesitate to taper medications due to concerns about relapse, while family members may either encourage discontinuation because of concerns about medication exposure or discourage it out of fear that symptoms will return.^54^ For these reasons, shared decision-making is central to deprescribing. Collaborative discussions regarding treatment goals, relapse risk, and tapering strategies can help align expectations and reduce anxiety surrounding medication discontinuation. Psychotherapeutic interventions, particularly cognitive-behavioral approaches, may further support this by strengthening coping strategies and reinforcing self-efficacy as medication is tapered.

In practice, the decision to stop or to continue pharmacotherapy in SAD must be individualized. For some, ongoing treatment provides stability and supports sustained functional recovery. For others who achieve durable remission and develop effective coping strategies, gradual deprescribing may be appropriate. Rather than viewing pharmacotherapy as either indefinite or time-limited, clinicians may instead conceptualize treatment as dynamic and iterative, with periodic reassessment of risks, benefits, and patient preferences. In this framework, deprescribing represents not the end of treatment but an integral component of the long-term management of anxiety disorders.

## Conclusion

The pharmacologic treatment of SAD has evolved from a limited set of options to a diverse and mechanistically heterogeneous armamentarium. Serotonergic treatment with either SSRIs or SNRIs remains the foundation of treatment, supported by the most evidence. However, across trials and in clinical practice, full remission is achieved in only a subset of patients,^22^^,^^143^ and many require sequential or combined strategies to achieve meaningful functional recovery.

Several practical clinical principles emerge from the available data. First, adequate dose and duration of first-line agents are essential, as premature discontinuation or subtherapeutic exposure may contribute to nonresponse, partial response, and early relapse.^143^ Second, the pharmacologic armamentarium should be viewed as a hierarchy rather than an interchangeable list: SSRIs and SNRIs are first-line; MAOIs and pregabalin are later-line but evidence-based; by contrast, antipsychotics, hormone-based approaches, and most novel mechanisms remain preliminary or investigational.^155^^,^^156^ Third, pharmacotherapy alone is rarely sufficient. While medications may reduce symptom burden and enable participation, they do not dismantle avoidance or safety behaviors—processes that require exposure-based learning.^20^^,^^157^ Fourth, in difficult-to-treat patients with persistent symptoms, targeted augmentation or alternative mechanisms should be considered, rather than repeated trials of similar agents.

Importantly, the treatment of SAD requires clinical equipoise. In other words, clinicians must balance risks with the potential consequences of undertreatment. While concerns regarding tolerability, dependence, or safety are appropriate, they should not lead to the systematic avoidance of effective interventions. In this regard, as reviewed herein, benzodiazepines and MAOIs, in particular clonazepam and phenelzine, have the highest effect size among SAD treatments (Cohen’s d ≈ 0.8–1).^37^ This is particularly relevant for patients with severe or treatment-resistant illness. However, their use is often complicated by clinician discomfort, access constraints, or monitoring requirements.^81^ Withholding these agents when clinically indicated may perpetuate suffering and functional impairment. In this context, thoughtful patient selection, careful monitoring, and shared decision-making are essential.

At the same time, the limitations of current treatments are clear. Adverse effects, delayed onset of action, challenges with adherence, and interindividual variability in pharmacokinetics and pharmacodynamics all contribute to persistent symptoms for many patients. There is a need to develop treatments that are more rapidly acting, better tolerated, and more precisely targeted to underlying neurobiologic mechanisms.

Emerging approaches, including chemosensory modulation and nicotinic receptor agonism, suggest that, in the future, therapeutics may move beyond traditional monoaminergic strategies, potentially offering more rapid or context-specific anxiolytic effects. However, these remain investigational, and their role in clinical practice is yet to be defined.

Ultimately, the goal of treatment in SAD extends beyond symptom reduction to restoration of function, social engagement, and quality of life. Achieving this requires a longitudinal, individualized approach that integrates pharmacologic and behavioral strategies, adapts to treatment response over time, and remains grounded in both evidence and clinical judgment. In this sense, pharmacotherapy for SAD is not a static algorithm, but a dynamic process that requires clinician and patient flexibility, persistence, and a willingness to use the full range of effective treatments when indicated, including both medications (the focus of this review) as well as psychotherapeutic interventions.

## Long descriptions

### Table 1. Long description

Seven survey respondents are labelled from A to G and their answers to five statements are shown, describing how far they agree or disagree. The statements were, I learnt something new, the tutor was engaging, I would recommend the course, I enjoyed the course, and I can apply what I learnt in my role. Four respondents answered positively to all the statements, selecting either Agree or Strongly Agree, one answered with either positive or neutral answers, and two answered all the statements with negative answers, selecting either Disagree or Strongly Disagree, plus one Neutral.

Navigate back to Table 1..

### Figure 1. Long description

Treatment algorithm beginning with CBT or an SSRI/SNRI. CBT responders continue treatment with gradual tapering; partial responders may receive other evidence-based psychotherapies, and nonresponders may receive an SSRI/SNRI. After inadequate response to an initial SSRI/SNRI, switch to another SSRI/SNRI, followed by consideration of pregabalin or a benzodiazepine and then an MAOI, preferably phenelzine. Partial responders may receive augmentation based on symptom pattern: PRN treatment for intermittent/provoked symptoms or maintenance treatment for persistent symptoms. Maintenance options include olanzapine, quetiapine, cariprazine, benzodiazepine, or CBT. Pharmacotherapy responders continue treatment for 12 months with reassessment every 3 months; persistent nonresponse may prompt combination strategies.

Navigate back to Figure 1..
